# Myopia awareness and knowledge among parents in Kumasi Metropolis and Bekwai Municipality

**DOI:** 10.4102/jphia.v16i1.1522

**Published:** 2025-10-15

**Authors:** Sylvester Kyeremeh, Percy K. Mashige, Kovin S. Naidoo

**Affiliations:** 1 Department of Optometry, Faculty of Health Sciences, University of KwaZulu-Natal, Durban, South Africa

**Keywords:** myopia, parental awareness, parental knowledge, refractive errors, parental perception, awareness creation, myopia awareness, myopia knowledge

## Abstract

**Background:**

Despite parents’ pivotal role in myopia mitigation, published studies investigating parental awareness and knowledge are limited in Ghana.

**Aim:**

Assess parental awareness and knowledge of myopia and related factors to mitigate myopia progression.

**Setting:**

Participants were parents from the Kumasi Metropolis and Bekwai Municipality in the Ashanti region of Ghana.

**Methods:**

A descriptive cross-sectional study was conducted using a semi-structured questionnaire. Participants were selected through a double-staged cluster sampling.

**Results:**

Of 747 participants, 500 (66.93%), reported no prior information about myopia, while 247 (33.07%) indicated awareness. Most of those aware (*n* = 182, 93.81%) demonstrated adequate knowledge. Predictors of awareness included male gender (odds ratio [OR] = 0.534, *p* = 0.023), training college (OR = 11.041, *p* < 0.001) and university education (OR = 21.536, *p* < 0.001), lower monthly income (Ghanaian cedi [Gh¢] 500.00 – Gh¢999.00; OR = 0.389, *p* = 0.038) and difficulty seeing afar (OR = 1.90, *p* = 0.023). Knowledge correlated with male gender (*p* = 0.036), monthly income (*p* < 0.001), type of work (*p* = 0.046) and age group (*p* = 0.042). Community-based approach was most preferred for myopia awareness creation.

**Conclusion:**

There was low myopia awareness but adequate knowledge levels, which significantly correlated with demographic factors. Community-based approach was the preferred myopia awareness creation mode.

**Contribution:**

The study provides insight into parental perspectives on myopia and reveals the preferred mode of myopia awareness and education in the Ghanaian context.

## Introduction

Myopia has become a public health concern globally, and its prevalence has seen a consistently increasing pattern, with the highest rates reported among Asian populations.^1,2^ Because of the significant global impact of myopia,^2^ various management strategies have been implemented to control its progression and prevent complications, thereby reducing its associated burden worldwide. For instance, among European children, the use of 0.01% atropine and defocus incorporated multiple segmented (DIMS) spectacles was found to be effective at reducing myopia progression and axial elongation.^3^ Positive lifestyle modifications, such as increased outdoor time, were reported to be protective against myopia development.^4^

Awareness and knowledge are critical factors influencing the adoption and implementation of myopia control strategies. While these terms are often used interchangeably because of their overlapping nature, they possess distinct definitions. Awareness refers to the ability to recognise or perceive the presence of a phenomenon or emerging trend.^5^ In contrast, knowledge denotes an understanding or familiarity with concepts, information, skills or phenomena, typically acquired through learning, experience or education.^6^ In relation to myopia, organisers of awareness creation efforts not only raise people’s awareness of the existence of myopia but also use the opportunity to educate them on various related topics, including definitions, symptoms, risk factors and management strategies.^7^

Developing adequate awareness and understanding of myopia is crucial for early identification of symptoms to facilitate timely interventions, identifying risk factors and promoting positive lifestyle modifications. In Ghana, public health institutions have been a key source of health information. An online survey among Ghanaian adults showed that for 41.9% of participants, the major sources of awareness were visits to hospitals, whereas 32.2% received their information from schools.^8^ For parents and guardians, in particular, knowledge about myopia is key because of their pivotal role in the lives of children. Parents and guardians spend a significant amount of time with children, play critical roles in their health and training^9^ and are likely to be the first to detect vision-related problems among them.^10^ The level of parental awareness and knowledge can significantly influence children’s acceptance and implementation of myopia management strategies.^11^ Furthermore, as childhood is the optimal period for introducing effective myopia control measures, parents play a critical role in ensuring these interventions’ successful adoption and outcomes.^12^

Several studies have documented varying levels of parental awareness and knowledge regarding different aspects of myopia. In Granada, Ortiz-Peregrina et al.^12^ reported that approximately 40% of parents were unaware of the existence of myopia control strategies and therefore, emphasised the need for increased dissemination of information to society, particularly to parents, about myopia, its consequences and available control options. Conversely, a study in Kenya reported high levels of myopia awareness and knowledge among parents; however, broader public awareness regarding the risks and corrective measures for myopia remained insufficient.^13^ Furthermore, in another study conducted in China, researchers identified a prevalent misunderstanding among parents regarding the role of insufficient outdoor activity in the development of myopia.^14^ The findings further indicated that parents often sought advice and acquired knowledge on myopia prevention only after their children had developed the condition.^14^ Finally, Qian and Lu,^15^ in a hospital-based study conducted in Suzhou, China, identified an adequate level of myopia-related knowledge among parents and guardians, which was attributable to their educational background. These research outcomes underscore the potential for targeted educational interventions for parents with the view to improving paediatric eye care outcomes, particularly myopia control strategies.

It is also important to understand the preferred means by which parents wish to receive myopia information. By incorporating such views into the design of myopia awareness programmes, with parents and guardians as primary targets, the acceptability of such interventions could be significantly enhanced. This highlights the need for intensified efforts to disseminate information aimed at reducing the risk and preventing the progression of myopia.

The Ashanti region is the second most populous among the 16 regions of Ghana, and it is administratively divided into 24 districts, 18 municipalities and the Kumasi Metropolis. The local governance act of Ghana defines district, municipality and metropolis as having a minimum population of 75 000, 95 000 and 250 000, respectively.^16^ Literacy rate in the region is 78%, being higher among males (82%) than females (74.2%).^17^ Among persons aged 15 years and older, the region contributes 18.2% to the national labour force, with key economic activities including service and sales, skilled agricultural, forestry and fishery workers.^17^

To the best of our knowledge, no published studies have specifically investigated parental awareness and knowledge of myopia in Ghana. A previous study, which aimed at assessing knowledge, attitude and preventive practices regarding myopia, focused on the general public rather than specifically targeting parents.^8^ Additionally, it employed an online-based survey, which potentially excluded people without Internet access and those who could not operate smart devices.^8^ Given parents’ pivotal role in managing myopia, understanding their awareness and knowledge and preferred mode of being informed about the condition, particularly in the African context, is essential. Therefore, the current study aimed to assess parental awareness and knowledge of myopia, identify factors influencing these outcomes and identify their preferred mode of receiving myopia information.

## Research methods and design

### Study design

An observational descriptive study design with cross-sectional sampling, using a semi-structured questionnaire, was employed, incorporating only participants available at the time of data collection. This methodology facilitated the examination of multiple outcome variables and was deemed appropriate because of its relative simplicity and alignment with the study’s time constraints.^18^

### Study setting and population

The study included parents and guardians residing in the Kumasi Metropolis and the Bekwai Municipality. The metropolis is the most populated administrative district within the Ashanti region, with a population size of 443 981, contributing 8.2% of the regional population. Occupying a land area of 68 square kilometres, the metropolis has the highest population density of 6542.6 persons per square kilometre.^17^ The urban nature of the metropolis is characterised with bustling business activities with service and sales being significantly notable. The Bekwai Municipality, with a population of 137 967, to a large extent, is rural and comprises 2.5% of the regional population size. The municipality covers a land area of 556 square kilometres, with a population density of 248.1 persons per square kilometre. Gender distribution within the two districts is comparable with females comprising 51.88% and 51.72% in the Kumasi Metropolis and Bekwai Municipality, respectively.^16^

### Sample size determination and sampling technique

The sample size was calculated using Cochran’s formula:


$$n=Z^{2}pq/e^{2},$$ [Eqn 1]

where *Z* is *z*-value for 95% confidence interval = 1.96, *p* is the estimated proportion of myopia in the population = 0.5 for maximum variability and *e* is the margin of error = 0.05, *q* = 1–*p*.

*n* = 1.96^2^ × 0.5 × 0.5 / 0.05^2^ = 384.16 ≈ 384.

Specific sample sizes were determined for each district (Kumasi Metropolis and Bekwai Municipality) by adjusting for each district’s population (based on) as follows:


$$n_{\times }=n/\left[1+(n-1)/N\right],$$ [Eqn 2]

where *n*_x_ is the specific sample size for the respective district, *n* is the calculated sample and *N* is the population for each district.

Therefore, for Kumasi Metropolis, the sample size, including a non-response rate of 10%, was 422, whereas that for the Bekwai Municipality, it was 421. Hence, the total sample size for both districts was 843.

A double-staged cluster sampling technique was used for participant selection. The clustering of the two districts was based on the Ghana Education (GES) Service’s organisational structure at the district level. This was because the research also included perspectives from basic school children. The findings from their responses have been reported in a separate publication. At the time of the study, there were 14 circuits in the Kumasi Metropolis, while the Bekwai Municipality had 10. The circuits in the Kumasi Metropolis were categorised into five clusters based on geographic proximity and demographic similarities. Similarly, the circuits in the Bekwai Municipality were grouped into three clusters using the same criteria. Subsequently, the participants were selected through random sampling, having obtained their informed consent to participate in the study.

### Eligibility criteria

Only participants who could understand the content of the questionnaire and could independently decide on the responses to give were included in the study. Participants who could not read the questionnaire independently or comprehend the questions, even after receiving guidance from trained research assistants, were excluded to avoid inaccurate responses.

### Data collection procedure and tools

A semi-structured questionnaire was developed and pretested to ensure reliability, achieving a Cronbach’s alpha of 0.70. The pretesting involved hand-delivery of the questionnaire to participants who shared similar demographic characteristics as the target population. These participants were not included in the actual study. Following pretesting of the questionnaire, it was refined by using clearer wording to ensure clarity. Final-year Doctor of Optometry students, who served as research assistants, received training to administer the questionnaires verbatim, particularly for participants who required assistance, thereby minimising potential biases and misinterpretations. The reliability of the instrument was further enhanced by selecting a suitable study population and including only items that were relevant to the research objectives.

The questionnaire was distributed to participants in person by the trained research assistants, who were available to provide guidance as needed. For parents or guardians who were not proficient in English, the research assistants facilitated the process by reading the questionnaire aloud in the local dialect to ensure accurate responses.

### Data management and statistical analysis

Data were entered into Microsoft^®^ Excel (version 27), cleaned and subsequently exported for statistical analysis using Stata (version 17) and SPSS^®^ (version 27). Open-ended responses were categorised and analysed quantitatively. Descriptive statistics, including frequency tables, central tendencies, percentages and graphical representations, were utilised. The Pearson chi-square test was used to assess the statistical significance of differences in observations. Binary logistic regression was performed to evaluate the influence of demographic factors on participants’ awareness of myopia. Myopia awareness was assessed by asking participants whether they had heard of myopia before. Participants who responded ‘yes’ were classified as being aware of myopia, while those who responded ‘no’ were classified as not being aware. The knowledge of participants who reported awareness of myopia was assessed using a 10-item Likert scale, with total scores ranging from 0 to 50. Participants achieving a score of 25 or higher were classified as having adequate knowledge, while those scoring below 25 were classified as having inadequate knowledge. An independent sample *t* test was conducted to compare mean myopia knowledge for independent variables with two groups, whereas a one-way analysis of variance (ANOVA) was utilised for independent variables with more than two groups. For the independent sample *t* test, effect size (Eta squared) was calculated from the formula:


$$\left[\text{Eta squared}=\left(t^{2}/t^{2}+(\text{N}1+\text{N}2-2\right)\right],$$ [Eqn 3]

where *t* is *t* test statistic, and N1 and N2 represent the sample sizes of the two groups. For ANOVA, the effect size was calculated as:


[Eta squared=sum of squares between groups/total sum of squares]. [Eqn 4]

Levene’s test was employed to assess variance equality (homogeneity). If homogeneity was assumed, the Tukey Honest Significant Difference (HSD) post hoc test was performed. In cases where homogeneity of variances was not assumed, the Welch test was applied, followed by the Games-Howell post hoc test. A *p*-value ≤ 0.05 was considered statistically significant for all inferential analyses.

### Ethical considerations

Ethical clearance to conduct this study was obtained from the Kwame Nkrumah University of Science and Technology Committee of Human Research, Publication and Ethics (No. CHRPE/AP/801/22) and the University of KwaZulu-Natal Humanities and Social Sciences Research Ethics Committee (No. HSSREC/00004822/2022). The study adhered to the provisions of the Declaration of Helsinki. Verbal consent was obtained from all participants prior to before their inclusion into the study. In order to ensure confidentiality and anonymity of participants, no personal information that could be traced to them was collected. Additionally, participants were assured that any information obtained from them would be used for research purposes only. Furthermore, data obtained were stored under lock for the duration of the study, with only the principal investigators having access. Participants were also assured that they were free to cease participating in the research whenever they wished without any consequences.

## Results

A calculated sample size of 843 was determined for the study; however, a total of 747 participants were enrolled, resulting in a response rate of 88.61%. Most participants were from the Kumasi Metropolis, with female participants comprising the larger proportion. The mean age of participants was 43.59 ± 10.65 years, with an age range of 20–83 years. Most participants fell within the 41- to 50-year group. The mean number of children per participant was 3.39 ± 1.65, with a range of 0–9 children. The majority reported Junior Secondary School (JSS), currently known as Junior High School (JHS), as their highest level of formal education. Approximately 10% of the participants indicated having no formal education. The most common highest educational qualification was a certificate, with about 5% of the participants possessing a master’s degree or higher.

The majority of participants were primarily involved in entrepreneurship or trade, with less than 1% employed in the engineering sector. Over 70% of the participants were self-employed, with the majority reporting a monthly income ranging from Ghanaian cedi Gh¢2000.00 – GH¢2999.00. Detailed demographic characteristics of the participants are presented in Table 1.

**TABLE 1:** Demographic information of participants.

Demographic information	Number of participants	Percentage
**Municipality (*N* = 747)**		
Bekwai	297	39.76
Kumasi	450	60.24
**Gender (*N* = 747)**		
Female	392	52.48
Male	355	47.52
**Age group (years) (*N* = 747)**		
20–30	94	12.58
31–40	224	29.99
41–50	234	31.33
51–60	149	19.95
61–70	40	5.35
≥ 71	6	0.80
**Number of children (*n* = 741)**		
0–2	237	31.98
3–5	417	56.28
6–8	87	11.74
**Highest education level (*n* = 742)**		
No formal education	74	9.97
Primary	33	4.45
JSS or JHS	253	34.10
SSS or SHS	171	23.05
Training college	61	8.22
University	150	20.22
**Highest qualification (*n* = 700)**		
None	85	12.14
Certificate	417	59.57
Diploma	51	7.29
Degree	109	15.57
Masters/PhD	38	5.43
**Work type (*n* = 723)**		
Banking or accounts	31	4.29
Business, entrepreneur or trading	353	48.82
Civil or security service	17	2.35
Artisans	80	11.07
Education or teaching	102	14.11
Driving	22	3.04
Agriculture related or farming	77	10.65
Catering	13	1.80
Health	12	1.66
Engineering (civil or electrical)	6	0.83
Retired or unemployed	10	1.38
**Employee type (*n* = 716)**		
Government employee	145	20.25
Self-employed	510	71.23
Private employee	54	7.54
Retiree	7	0.98
**Monthly income (Gh¢) (*n* = 627)**		
< 500.00	93	14.83
500.00–999.00	115	18.34
1000.00–1999.00	138	22.01
2000.00–2999.00	173	27.59
3000.00–3999.00	54	8.61
4000.00–4999.00	19	3.03
≥ 5000.00	35	5.58

SSS, Senior Secondary School; SHS, Senior High School; JSS, Junior Secondary School; JHS, Junior High School.

A total of 544 participants (73.12%) reported no difficulty with distance vision, while 200 participants (26.88%) reported experiencing difficulty with distance vision.

### Myopia awareness

Participants were asked whether they had heard about myopia. A majority, 500 (66.93%), reported no prior knowledge, while 247 (33.07%) indicated awareness. Most of those unaware of myopia resided in the Kumasi Metropolis (*n* = 328, 65.6%). Conversely, slightly more than half of those who were aware of myopia were from the Bekwai Municipality (*n* = 125, 50.61%), compared to 122 (49.39%) from the Kumasi Metropolis. This distribution was statistically significant ($\chi_{\left(1747\right)}^{2}$ = 18.132, *p* < 0.001) as shown in Table 2.

**TABLE 2:** Participants’ responses on awareness of myopia compared with participants’ district and distance vision status.

Factor	Heard about myopia	Not heard about myopia	*χ* ^2^	*df*	*P*
Kumasi	122	328	18.132	1747	0.000*
Bekwai	125	172	-	-	-
Difficulty seeing at far	72	128	1.065	1744	0.302
No difficulty seeing at far	174	370	-	-	-

*df*, degree of freedom.

* , Statistically significant.

Additionally, the majority of participants who reported difficulty with distance vision were unaware of myopia compared to those without such difficulty. However, this difference was not statistically significant (χ^2^ = 1.065, *p* = 0.302), as illustrated in Table 2.

### Source of information on myopia

**FIGURE 1:**
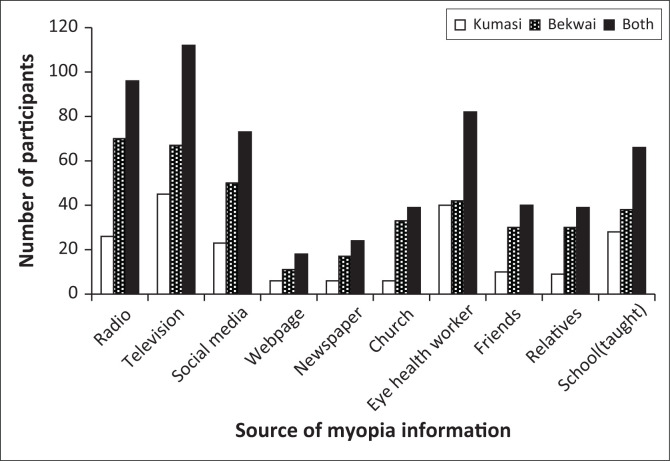
Participants’ responses on source of myopia information compared to participants’ district.

A total of 589 multiple responses were recorded from 216 participants regarding the sources of information about myopia. Television was the most frequently reported source, accounting for 112 (19.00%), followed by radio with 96 (16.30%) responses. In contrast, web pages were the least reported source, with only 18 (3.10%) responses, as detailed in Figure 1. Radio was the primary source of information among participants in the Bekwai Municipality, while television was the predominant source in the Kumasi Metropolis. In both districts, web pages represented the least utilised source of information regarding myopia as shown in Figure 1.

### Participants’ perspective on myopia awareness level within their communities

**FIGURE 2:**
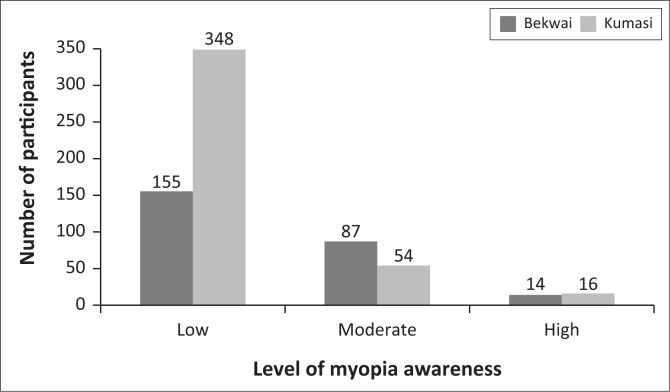
Perceived level of myopia awareness compared with participants’ district.

Among the 674 participants who answered questions regarding the level of myopia awareness among parents in their communities, 503 (74.63%), 141 (20.92%) and 30 (4.45%) perceived awareness to be low, moderate and high, respectively. A Pearson Chi-square test revealed a significant association between participants’ districts and their perception of myopia awareness levels ($\chi_{\left(2674\right)}^{2}$ = 45.61, *p* < 0.001). Notably, the majority of those who reported low levels of myopia awareness were from the Kumasi Metropolis, whereas most participants reporting moderate levels of myopia awareness were from the Bekwai Municipality (Figure 2).

### Predicting factors for myopia awareness among participants

A binary logistic regression analysis was conducted to determine the association between demographic factors and perceived myopia awareness among the study participants. The model, which included all demographic predictors, was statistically significant ($\chi_{\left(33,\;n\;=\;593\right)}^{2}$ = 274.85, *p* < 0.001), accounting for 37.10% of the variance in myopia awareness levels as per Cox and Snell’s *R*^2^ and 51.30% as per Nagelkerke *R*^2^. Significant predictors of myopia awareness included gender, highest education level, monthly income and reported distance vision status (Online Appendix 1 Table 4-A1). Males were approximately 0.5 times less likely than females to have heard about myopia (OR [odds ratio] = 0.534, 95% CI [confidence interval]: 0.311–0.916, *p* = 0.023). Participants with a college education were about 11 times more likely to report awareness of myopia compared to those without formal education (OR = 11.041, 95% CI: 2.956–41.241, *p* < 0.001). Similarly, individuals with university-level education were approximately 22 times more likely to have heard about myopia compared to those with no formal education (OR = 21.536, 95% CI: 6.187–74.960, *p* < 0.001).

Participants with a monthly income of less than Gh¢500.00 (< $34.03) were used as the reference group in the analysis. Compared to this group, individuals with a monthly income of Gh¢500.00 – Gh¢999.00 (OR = 0.389, 95% CI: 0.165–0.917, *p* = 0.038), Gh¢1000.00 – Gh¢1999.00 (OR = 0.299, 95% CI: 0.119–0.748, *p* = 0.010), Gh¢2000.00 – Gh¢2999.00 (OR = 0.157, 95% CI: 0.059–0.421, *p* < 0.001) and Gh¢3000.00 – Gh¢3999.00 (OR = 0.216, 95% CI: 0.066–0.712, *p* = 0.012) were less likely to have heard about myopia. Additionally, individuals who reported difficulty seeing at a distance were approximately twice as likely to have heard about myopia compared to those without such difficulties (OR = 1.90, 95% CI: 1.091–3.309, *p* = 0.023). Further details of the logistic regression analysis are provided in Online Appendix 1 Table 4-A1.

### Participants’ perspectives on myopia education

Participants were asked whether there was a need for the development of a myopia awareness programme. Of the 714 participants who responded, 646 (90.48%) agreed that there should be such a programme while 68 (9.52%) thought otherwise.

Regarding the mode of programme for creating myopia awareness, 217 (36.78%) participants preferred a community-based approach, while 143 (24.24%) recommended the use of television, radio or other media. Table 3 provides further detail on the participants’ preferred mode of myopia awareness creation.

**TABLE 3:** Participants’ preferred mode of myopia awareness programme.

Mode of programme	Number of participants	Percentage
Community based	217	36.78
Through parent-teacher-associations	30	5.08
House-to-house	10	1.69
Taught in school	50	8.47
Through screening or outreach	68	11.53
Television, radio or media	143	24.24
Through eye clinics	14	2.37
Workplace based	24	4.07
Through the information centre	34	5.76

**Total**	**590**	**100**

### Myopia knowledge

A total of 194 participants responded to questions on myopia knowledge, yielding a mean score of 34.77 ± 6.30 (95% CI: 33.88–35.66), with scores ranging from 15 to 48. The Shapiro-Wilk test of normality indicated that the data were approximately normally distributed (*W*[194] = 0.988, *p* = 0.098), with skewness (standard error) = –0.346 (0.175) and kurtosis (standard error) of 0.121 (0.347). The mean myopia knowledge scores stratified by demographic factors are presented in Table 4.

**TABLE 4:** Distribution of mean myopia knowledge scores over demographic information.

Demographic	Sample (*n*)	Mean	s.d.	95% CI	
				Lower bound	Upper bound
**Municipality**					
Bekwai	95	34.46	6.90	33.06	35.87
Kumasi	99	35.06	5.70	33.92	36.29
Total	194	34.77	6.30	33.88	35.66
**Gender**					
Female	104	33.88	6.82	32.56	35.21
Male	90	35.79	5.52	34.63	36.94
Total	194	34.77	6.30	33.88	35.66
**Distance vision status**					
With difficulty	60	35.85	5.89	34.33	37.37
Without difficulty	133	34.32	6.46	33.20	35.42
Total	193	34.79	6.31	33.90	35.69
**Age group (years)**					
20–30	37	32.00	7.36	29.55	34.45
31–40	78	35.97	5.47	34.74	37.21
41–50	52	34.25	5.90	32.61	35.89
51–60	21	35.52	7.25	32.22	38.82
61–70	6	38.00	4.70	33.08	42.92
Total	194	34.77	6.30	33.88	35.66
**Number of children**					
0–2	81	35.19	6.25	33.80	36.57
3–5	100	34.57	6.48	33.29	35.85
6–8	12	34.17	5.46	30.70	37.63
Total	193	34.80	6.30	33.91	35.70
**Employment type**					
Government employee	87	36.01	5.92	34.75	37.27
Self-employed	75	33.96	6.63	32.43	35.49
Private employee	24	33.04	6.82	30.16	35.92
Retiree	2	38.00	0.00	38.00	38.00
Total	188	34.84	6.38	33.92	35.75
**Highest education level**					
No formal education	7	32.00	4.16	28.15	35.85
Primary	4	31.00	7.07	19.75	42.25
JSS or JHS	30	34.33	6.84	31.78	36.89
SSS or SHS	23	32.22	6.90	29.23	35.20
Training college	36	35.83	6.76	33.55	38.12
University	93	35.56	5.73	34.38	36.74
Total	193	34.80	6.31	33.90	35.69
**Monthly income (Gh¢)**					
< 500	23	30.57	6.91	27.58	33.55
500.00–999.00	18	32.17	5.53	29.42	34.92
1000.00–1999.00	40	35.67	5.60	33.88	37.47
2000.00–2999.00	47	37.28	6.31	35.42	39.13
3000.00–3999.00	20	36.70	5.07	34.33	39.07
4000.00–4999.00	13	34.15	6.26	30.37	37.93
≥ 5000.00	19	33.74	6.24	30.73	36.75
Total	180	34.89	6.36	33.95	35.82
**Work type**					
Banking or accounts	20	35.10	4.08	33.19	37.01
Business, entrepreneur or trading	49	34.63	5.90	32.94	36.33
Civil or security service	11	37.18	5.88	33.23	41.13
Artisans	15	32.47	6.73	28.74	36.19
Education or teaching	62	35.73	6.34	34.11	37.34
Driving	3	36.33	3.06	28.74	43.92
Agriculture related or farming	14	29.71	6.88	25.74	33.69
Catering	2	34.50	2.12	15.44	53.56
Health	8	36.00	5.45	31.44	40.56
Engineering (civil or electrical)	4	29.25	11.87	10.36	48.14
Retired or unemployed	3	36.67	2.31	30.93	42.40
Total	191	34.65	6.25	33.76	35.55

s.d., standard deviation; CI, confidence interval; SSS, Senior Secondary School; SHS, Senior High School; JSS, Junior Secondary School; JHS, Junior High School.

### Comparison of mean myopia knowledge scores over demographic information

Independent sample *t* test with equal variances of variables.

An independent sample *t* test was conducted to determine whether mean myopia knowledge scores differed significantly by district, gender and distance vision status. Levene’s test for equality of variances indicated homogeneity of variances across all variances: district (*F* = 3.684, *p* = 0.056), gender (*F* = 2.442, *p* = 0.120) and distance vision status (*F* = 1.150, *p* = 0.285). The results revealed that the mean score for females was significantly lower than that for males (*t*[192] = –2.117, *p* = 0.036), with a small effect size of Cohen’s *d* = 0.02. No significant differences were observed for district (*t*[192] = –0.659, *p* = 0.511) or distance vision status (*t*[191] = –1.569, *p* = 0.118). Detailed results are presented in Table 3.

### One-way analysis of variance with equal variances

A one-way ANOVA was conducted to assess significant differences in mean myopia knowledge scores based on participants’ monthly income and type of work. Levene’s test for equality of variances indicated the homogeneity of variances (*p* = 0.776). The analysis revealed statistically significant differences for both monthly income (*F*_[6173]_ = 4.378, *p* < 0.001) and type of work (*F*_[10 180]_ = 1.909, *p* = 0.046), with medium effect sizes of 0.13 and 0.10, respectively, as presented in Table 4. Post hoc analysis using Tukey HSD test indicated that the mean myopia knowledge scores for individuals with a monthly income of < Gh¢500.00 were significantly different from those earning Gh¢1000.00 – Gh¢1999.00 (*p* = 0.024), Gh¢2000.00 – Gh¢2999.00 (*p* < 0.001), and Gh¢3000.00 – Gh¢3999.00 (*p* = 0.018). Additionally, a significant difference was observed between those earning Gh¢500.00 – Gh¢999.00 and Gh¢2000.00 – Gh¢2999.00 (*p* = 0.040). Regarding the type of work, significant differences were found between participants in agriculture-related occupations and those in education/teaching-related work (*p* = 0.041). Further details of the post hoc analysis are provided in Online Appendix 1, Table 1-A1 and Table 2-A1.

### Analysis of variance with unequal variances assumed

An ANOVA was conducted to determine significant differences in the mean myopia knowledge scores across the variables of age group, number of children, type of employment and highest level of education. Levene’s test for homogeneity of variances indicated that the assumption of equal variances was violated (*F*_[4189]_ = 2.739, *p* = 0.030). Consequently, the Welch ANOVA was utilised, revealing a statistically significant difference between groups (*W*_[430.94]_ = 2.820, *p* = 0.042). Post hoc analysis using the Games-Howell test identified a significant difference in myopia knowledge between the age groups 20–30 years and 31–40 years (*p* = 0.038), as shown in Online Appendix 1, Table 3-A1. No significant differences were found for number of children (*F*[2190] = 0.276, *p* = 0.759), type of employment (*F*[3184] = 2.299, *p* = 0.079) or highest level of education (*F*[5187] = 1.875, *p* = 0.101).

**FIGURE 3:**
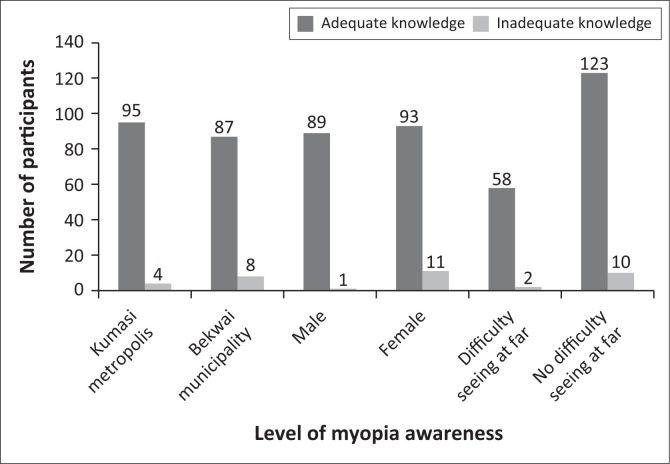
Participants’ level of myopia knowledge.

Of the 194 participants who responded to myopia knowledge questions, 182 (93.81%) demonstrated adequate knowledge, while 12 (6.19%) exhibited inadequate knowledge. Fischer’s exact test showed a significant difference in the level of knowledge between male and female participants (*p* = 0.006). However, no significant differences were observed in knowledge levels between participants from Kumasi Metropolis and Bekwai Municipality (*p* = 0.244), nor between individuals with and without difficulty seeing from afar (*p* = 0.348) (Figure 3).

## Discussion

The current study sought to assess the level of awareness and knowledge of myopia among parents and guardians in the Bekwai Municipality and Kumasi Metropolis in the Ashanti region of Ghana. It also sought to determine their perspectives on the mode of myopia awareness creation. The results revealed a low level of myopia awareness among participants. However, most of those who were aware of myopia demonstrated adequate knowledge of the condition. Regarding the mode of myopia awareness creation, participants reported that a community-based approach was preferred to other methods. These results highlight the critical need to improve myopia education through targeted awareness campaigns. Based on this evidence, effective strategies for enhancing myopia education can be developed. Furthermore, significant associations were observed between certain demographic factors and myopia awareness and knowledge levels. These associations enhance the understanding of factors influencing myopia awareness and also help to identify key considerations for designing effective myopia awareness interventions.

Two-thirds of participants reported no prior knowledge of myopia, highlighting the low level of awareness about the condition. Participants also perceived myopia awareness within their communities to be minimal. This suggests a general lack of understanding of myopia risks and complications. The limited awareness may stem from insufficient public education initiatives or cultural and linguistic barriers, as the term ‘myopia’ lacks a direct equivalent in Twi, the predominant language in the study area, potentially hindering comprehension and relatability. Similarly, a 2024 survey on parental awareness in the UK revealed that while many parents understood myopia as ‘short-sightedness’, fewer were familiar with the specific term ‘myopia’.^19^ This underscores the need to consider linguistic and language issues as critical factors, which could influence awareness creation interventions. Given the critical role of parents and guardians in implementing myopia-related preventive and management strategies as they interact with children – the primary at-risk group – over extended periods, addressing factors that could influence their understanding of health-related issues, such as myopia, is key.^15^ The low myopia awareness among participants could impact the health-seeking behaviour of the children, as insufficient parental awareness of myopia may reduce the perceived urgency to seek eye care, thereby causing delays in obtaining treatment for affected children.^20^ Therefore, parental understanding and acceptance of lifestyle modifications and other myopia control interventions may be adversely affected, as these depend on parental awareness.^21^ A Chennai, India, study reported that parents’ perceptions and awareness were pivotal in determining whether to seek appropriate eye care for children.^11^ Our findings highlight the urgent need for intervention strategies to enhance myopia awareness among parents and/or guardians.

The comparison of myopia awareness between the two districts revealed that the proportion of individuals aware of the condition was slightly higher in Bekwai Municipality than in the Kumasi Metropolis, with this difference being statistically significant (Table 2). Typically, urbanised communities are expected to exhibit higher levels of myopia awareness because of the condition’s higher prevalence in such settings compared to suburban areas.^22^ However, an interesting anomaly was observed in this study. At the time of data collection, a school screening programme organised by a non-governmental organisation was actively identifying refractive errors within the Bekwai Municipality. It is plausible that this initiative could have influenced the findings of this study, as such programmes have been reported to enhance awareness of myopia and other vision-related disorders.^23^

Participants who were aware of myopia predominantly sourced their information from television and radio, with web pages being the least utilised medium. This trend may be attributed to the widespread availability and accessibility of television and radio compared to the relatively low household Internet penetration. According to a recent survey by the National Communications Authority, 68.9% and 70.1% of Ghanaian households owned televisions and radios, respectively, while only 16.8% had Internet access.^24^ Furthermore, as the majority of participants had attained only junior secondary or high school education, television and radio may be more convenient and accessible than web pages, which require a higher level of technological proficiency. Television and radio are widely accepted and user-friendly platforms, and with advancements in technology, many smartphones now incorporate television and radio functions, offering users diverse options for accessing information. While younger individuals may demonstrate a preference for social media and other modernised sources of information,^25,26^ our findings suggest the potential value of incorporating television and radio as critical components in designing myopia awareness interventions targeted at parents. Contrary to the current study’s findings, Ortiz-Peregrina et al.^12^ in an online survey of parents who had children with myopia in Granada, Spain, identified relatives and eye care practitioners as the primary sources of myopia-related information. Similarly, a population-based online study of adults aged 18 years and above in Ghana reported that hospitals and schools served as the main sources of information on myopia.^8^

Awareness of myopia was significantly associated with gender, with males being less likely to have heard about myopia compared to females. Evidence suggests a gender predisposition towards females in myopia prevalence, as noted in prior studies.^27,28,29^ The higher prevalence observed among females may influence their eye health-seeking behaviour, potentially contributing to greater awareness of the condition.

Additionally, educational background was significantly associated with myopia awareness, with individuals possessing higher levels of education being more likely to have knowledge of the condition. Compared to participants with no formal education, those whose highest level of education was training college were 11 times more likely to have heard about myopia, while those with a university education were about 22 times more likely. This finding underscores the critical role of formal education in enhancing myopia awareness. The importance of education in promoting myopia awareness is further supported by Huang et al.^20^ who demonstrated that school-based myopia prevention education significantly improves knowledge, attitudes and skills related to myopia among Chinese middle school students. This highlights the potential of educational interventions, even at lower levels, in fostering awareness and preventive practices for myopia.

Increasing monthly income was associated with reduced odds of being aware of myopia. Compared to participants earning less than Gh¢500.00 (approximately $34.00) per month, individuals earning Gh¢500.00 – Gh¢999.00, Gh¢1000.00 – Gh¢1999.00, Gh¢2000.00 – Gh¢2999.00 and Gh¢3000.00 – Gh¢3999.00 were, respectively, 0.4, 0.3, 0.2 and 0.2 times as likely to have heard about myopia. Although it is generally expected that higher-income individuals would have greater access to education, healthcare services and information, leading to better awareness, this was not observed in the current study. Further research is warranted to explore the underlying factors contributing to this trend. Nonetheless, the findings highlight a significant association between socio-economic status and myopia awareness.

Participants with difficulty seeing at a distance were approximately twice as likely to have heard about myopia compared to those without visual difficulties. Visual status may influence health-seeking behaviours, as individuals with visual impairments are more likely to seek care. Visits to healthcare facilities have been reported as a key source of health information.^8^

Myopia knowledge was assessed exclusively among the one-third of participants who reported awareness of the condition. Among these individuals, the majority (93.81%) demonstrated adequate knowledge. However, this subgroup accounted for only 24.36% of the total sample, indicating an overall low level of myopia knowledge among the study population. These findings differ from the population-based online study in Ghana, which reported a higher proportion of participants with adequate myopia knowledge.^8^ In that study, approximately 56% of participants had completed tertiary education, whereas only 28% of participants in the current study possessed a similar level of education.^8^

There was a significant association between myopia knowledge and gender, type of work, age group and monthly income. Female participants demonstrated slightly lower knowledge scores compared to males (Table 3), with the difference being minimal and reflected by a small effect size of 2.00%. However, a similar study conducted in Kenya reported that women exhibited greater knowledge of myopia risk factors compared to men.^13^

Participants involved in education or teaching-related professions had significantly higher knowledge scores than those involved in agriculture-related occupations, such as farming. This disparity may be attributed to higher levels of education and increased access to information among individuals in white-collar professions, which generally contributes to greater awareness of myopia risk factors compared to those in blue-collar occupations.^13^ As reported by other researchers, the significant association between occupational type and knowledge scores aligns with evidence demonstrating variability in health literacy levels across different occupational groups.^10^

Regarding age, a significant difference was observed between the 20- to 30-year and 31- to 40-year age groups, with the latter exhibiting higher knowledge scores. This finding contrasts with expectations, as younger individuals are presumed to have greater access to diverse information sources, including social media and other Internet-based platforms.^8^ The observed outcome may be attributable to a higher proportion of highly educated participants within the 31- to 40-year age group. However, the Ghanaian online study among adults aged 18 years and above found a negative correlation between myopia knowledge and age.^8^ These discrepancies in myopia knowledge across demographic factors highlight the importance of implementing targeted, rather than generalised, interventions to enhance awareness and knowledge about myopia.

Participants mainly received myopia information from television, radio and through eye health workers. The findings emphasise the critical role of traditional media as well as eye health workers in the creation of myopia awareness. These media are reported to be highly accessible, affordable and can be tailored to local contexts, which makes them effective means for the dissemination of information about issues that are relevant to society.^30^ Eye health workers are key stakeholders equipped with both the knowledge and skills that are relevant for the mitigation of myopia. Hence, most myopia advocacy efforts emphasise the significant role of the eye health workers in raising myopia awareness and fostering positive lifestyle modifications. In contrast to the findings of the current study, Ortiz-Peregrina et al.’s online survey among parents who had myopic children found relatives and eye care practitioners to be the main sources of myopia information.^12^

Regarding the choice of modality to be used for the development of a myopia awareness creation programme, participants mainly preferred a community-based approach, which was followed by the use of traditional media, such as television and radio. Buttressing the relevance of community-based health education programmes, Blessing^31^ acknowledges that such programmes play a vital role in improving public health by empowering individuals and communities with knowledge and resources to make informed health choices. Hence, considering the choice of participants in the current study, the development of myopia education programmes could ensure appropriateness. However, Merzel and D’Afflitti^32^ in a systematic literature review of 32 community-based prevention programmes in the United States since 1980 report that such interventions only provide modest impact owing to factors such as methodological challenges to study design and evaluation, concurrent secular trends, among others. Large-scale community-based health promotion programmes, for example, are reported to often result in limited or missing population-wide changes because of the aforementioned challenges.^33^ Notwithstanding these inconsistencies regarding the impact of community-based health promotion interventions, in a worldwide systematic literature review on community-based health promotion and prevention programmes, Nickel and Knesebeck found results confirming that such interventions are promising for health promotion and disease prevention, but their potential is not fully realised.^33^ Therefore, in the current study, although participants largely preferred a community-based approach for myopia awareness creation, further studies to identify the most appropriate strategy to ensure a significant post-intervention outcome are warranted.

### Strengths and limitations of the study

The strengths of the study lie in the large sample size and the rigorous statistical analysis employed. Additionally, the design enabled the examination of relationships among multiple outcome variables, which provided a broad overview of the study characteristics relevant to the aim of the research. Finally, the relative simplicity of the design and its alignment with the study’s time constraints provided a significant opportunity to obtain critical population information that is relevant for public health intervention and future studies. However, the study has some limitations, which have to be taken into account when interpreting the results. To begin with, the relatively smaller response rate for some questions may potentially introduce selection and non-response bias, which could influence the generalisability of the findings. Again, the study could not establish temporal and causal relationships because of its cross-sectional nature. Furthermore, the study did not specifically include parents of children with myopia and therefore, the prevalence-based sample size may not fully represent the general parent population. Finally, the use of a survey may potentially introduce recall bias, which may result in drawing inaccurate conclusions.

### Recommendation

Based on the outcome of the study, the authors recommend that future projects should highlight interventions that focus on raising myopia awareness and knowledge, with parents as primary targets. Additionally, further research is warranted to exhaustively explore the relationships between demographic factors and myopia awareness with the view to informing public health policy and programmes.

## Conclusion

The findings of the study indicate a low level of myopia awareness among participants, with television and radio being the main sources of information. Notably, those who were aware of the condition demonstrated adequate knowledge about it. Awareness of myopia was significantly associated with gender, highest educational attainment, monthly income and distance vision status. Knowledge of myopia was significantly associated with gender, type of work, age group and monthly income. Participants cited community-based approach as their preferred mode for myopia awareness creation. These findings underscore the urgent need for targeted myopia education interventions, particularly among parents, given their pivotal role in myopia mitigation. Furthermore, the findings highlight commonly utilised as well as preferred information sources, which could be strategically integrated into future educational initiatives.
